# Clinical implications of PD‐L1 expression in oncogene‐driven NSCLC: Differential responses to targeted agents and immune checkpoint inhibitors

**DOI:** 10.1002/ijc.70413

**Published:** 2026-03-23

**Authors:** Xiaoxiao Fan, Chenxi Wei, Minjun Rong, Shengnan Wang, Jiaying Wang, Xiaohan Wang, Xiao Han, Xue Meng

**Affiliations:** ^1^ Shandong Cancer Hospital and Institute Shandong First Medical University and Shandong Academy of Medical Sciences Jinan Shandong China; ^2^ Department of Medical Oncology Huantai County People's Hospital Zibo Shandong China; ^3^ Family & Women‐Children Health & Family Planning Division Health and Wellness Service Center (Center for Disease Control and Prevention) of Binzhou Economic and Technological Development Zone Binzhou Shandong China; ^4^ School of Public Health Shandong First Medical University & Shandong Academy of Medical Sciences Jinan China

**Keywords:** driver gene mutations, immunotherapy, non‐small cell lung cancer (NSCLC), PD‐L1 expression, tyrosine kinase inhibitors (TKIs)

## Abstract

PD‐L1 is a reliable biomarker for predicting immunotherapy efficacy in NSCLC patients without driver gene mutations. However, its significance in patients with driver gene‐positive tumors remains unclear. This study analyzed 273 patients with stage IV non‐small cell lung cancer harboring driver gene mutations. All patients had PD‐L1 expression levels ≥10%. The effect of PD‐L1 expression on progression‐free survival (PFS) and overall survival (OS) was assessed. Among the 273 patients with driver gene‐positive NSCLC, 127 had a PD‐L1 tumor proportion score (TPS) of 10–49%, and 146 had TPS ≥50%. Patients in the TPS 10%–49% group had significantly better median PFS and OS compared to those in the TPS ≥50% group (*p* = 0.0008 and *p* = 0.0009, respectively). In the realm of targeted therapy, patients who received first‐line tyrosine kinase inhibitors (TKIs) showed superior outcomes in the TPS 10–49% group compared to the TPS ≥50% group in terms of both median PFS (30.8 months vs. 13.9 months, *p* = .0001) and OS (44.8 months vs. 26.3 months, *p* = .0006). Regarding immunotherapy, PD‐L1 expression level was not significantly associated with treatment efficacy. However, in patients with KRAS mutations, those who received first‐line immunotherapy exhibited better median PFS and OS (*p* <.0001 for both). Notably, among patients with high PD‐L1 expression, no statistically significant clinical benefit was observed. In NSCLC patients with driver gene mutations, PD‐L1 expression is associated with the efficacy of targeted therapy but is not predictive of response to immunotherapy.

## What's New?

PD‐L1 is a reliable biomarker for predicting immunotherapy efficacy in patients with non‐small‐cell lung cancer lacking oncogenic driver mutations. However, its significance in patients with driver mutations remains unclear. In this study of 273 patients with stage IV non–‐small‐cell lung cancer harboring diverse driver alterations and PD‐L1 tumor proportion scores ≥ 10 %, high PD‐L1 expression predicted shorter progression‐free and overall survival after first‐line tyrosine‐kinase inhibitors, but not immunotherapy response. The authors proposed a nomogram integrating PD‐L1, age, and histology for individualized 1‐ to 3‐year survival estimates, offering a practical tool to optimize treatment selection in oncogene‐driven disease.

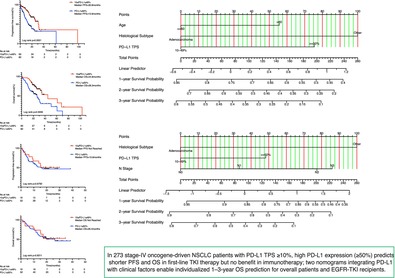

**Abbreviations**
AJCC: American Joint Committee on Cancer
ALK: anaplastic lymphoma kinase
BRAF: v‐raf murine sarcoma viral oncogene homolog B1
ECOG: Eastern Cooperative Oncology Group
EGFR: epidermal growth factor receptor
EGFR 19Del: EGFR exon 19 deletion
EGFR 21L858R: EGFR exon 21 L858R point mutation
FFPE: formalin‐fixed paraffin‐embedded
HER2: human epidermal growth factor receptor 2
ICI: immune checkpoint inhibitor
IHC: immunohistochemistry
KRAS: Kirsten rat sarcoma viral oncogene homolog
KRAS‐G12C: KRAS codon 12 glycine to cysteine substitution
MET: mesenchymal–epithelial transition factor
METamp: MET gene amplification
METex14: MET exon 14 skipping mutation
N: node
NRAS: neuroblastoma RAS viral oncogene homolog
NSCLC: non‐small‐cell lung cancer
OS: overall survival
PD‐L1: programmed death‐ligand 1
PFS: progression‐free survival
PIK3CA: phosphatidylinositol‐4, 5‐bisphosphate 3‐kinase catalytic subunit alpha
RECIST: response evaluation criteria in solid tumors
RET: rearranged during transfection
ROS1: c‐ros oncogene 1
T: tumor
TKI: tyrosine kinase inhibitor
TPS: tumor proportion score

## 1 INTRODUCTION

Lung cancer remains the leading cause of cancer‐related morbidity and mortality worldwide. Non‐small‐cell lung cancer (NSCLC) accounts for approximately 85% of all lung cancer cases, and the majority of patients present with metastatic disease at diagnosis, contributing to a dismal 5‐year relative survival rate of approximately 10%.^1^, ^2^, ^3^ In patients with NSCLC lacking actionable driver mutations, immune checkpoint inhibitors (ICIs), either as monotherapy or in combination with chemotherapy, have become the standard of care in both first‐line and subsequent treatment settings.^4^, ^5^, ^6^ Programmed death‐ligand 1 (PD‐L1) expression has emerged as a widely accepted biomarker predictive of response to ICIs, with higher expression levels generally correlating with improved therapeutic efficacy.^7^, ^8^

In contrast, the role of PD‐L1 expression in patients with oncogenic driver mutations remains uncertain. Although targeted therapies, such as tyrosine kinase inhibitors (TKIs), have led to substantial improvements in clinical outcomes among patients harboring driver mutations, resistance to these agents remains a major therapeutic challenge.^9^, ^10^, ^11^, ^12^, ^13^ Emerging evidence suggests that PD‐L1 expression may be associated with intrinsic or acquired resistance to targeted therapies in this population.^14^, ^15^, ^16^, ^17^ This overlap raises important questions regarding the prognostic and predictive utility of PD‐L1 in the context of concurrent oncogenic alterations.

In this study, we investigated the impact of PD‐L1 expression on treatment outcomes in patients with advanced NSCLC harboring driver mutations. Given emerging evidence that higher PD‐L1 TPS thresholds may better capture prognostic heterogeneity in oncogene‐driven disease, we focused on patients with PD‐L1 TPS ≥10% and categorized them into moderate (TPS 10%–49%) and high (TPS ≥50%) expression groups.^14^, ^18^, ^19^ We assessed the association between PD‐L1 expression and clinical outcomes following targeted therapy and immunotherapy, and developed a nomogram integrating independent prognostic factors to enable individualized risk prediction.

## 2 METHODS

### 2.1 Patients and data collection

We conducted a retrospective study of patients with advanced NSCLC diagnosed and treated at the Cancer Hospital of Shandong First Medical University between November 2020 and September 2022. Inclusion criteria were a confirmed diagnosis of stage IV NSCLC with both driver gene alterations and PD‐L1 expression, as verified by pathological reports. Only patients with a tumor proportion score (TPS) for PD‐L1 of ≥10% were included.

A total of 273 patients met the eligibility criteria. Clinical and demographic characteristics—including sex, age, Eastern Cooperative Oncology Group (ECOG) performance status, histological subtype, smoking status, oncogenic driver mutation profile, stage, metastatic status and PD‐L1 expression level—were collected from medical records. Tumor stage was assessed according to the 8th edition of the American Joint Committee on Cancer (AJCC) TNM classification system.^20^ Treatment response was evaluated using the Response Evaluation Criteria in Solid Tumors (RECIST), version 1.1.^21^

### 2.2 PD‐L1 immunohistochemistry

PD‐L1 protein expression was evaluated by immunohistochemistry (IHC) using the PD‐L1 IHC 22C3 pharmDx assay (clone 22C3, Agilent Technologies). Formalin‐fixed, paraffin‐embedded (FFPE) tissue specimens were used to prepare 4 μm‐thick sections. Immunohistochemical staining was performed strictly according to the manufacturer's instructions on the Dako Autostainer Link 48 fully automated IHC staining platform, using the 22C3 anti‐PD‐L1 monoclonal antibody. The TPS was defined as the percentage of viable tumor cells exhibiting partial or complete membrane staining at any intensity relative to the total number of viable tumor cells. Necrotic areas, stromal cells, and tumor‐infiltrating immune cells were excluded during evaluation. All slides were independently assessed by two experienced pathologists who were blinded to the patients' clinical outcomes. Any discrepant interpretations were resolved through a joint review to reach a consensus. All detections were conducted in the Department of Pathology, Shandong Cancer Hospital Affiliated to Shandong First Medical University, which has obtained relevant quality control certification.

The tissue specimens used for PD‐L1 detection in this study included primary lung tumor tissues (*n* = 202, 73.99%) and metastatic tissues (*n* = 71, 26.01%) (Table 1). The specific anatomical sites of the metastatic tissues were as follows: lymph nodes (*n* = 35), bone (*n* = 8), liver (*n* = 6), adrenal gland (*n* = 5), brain (*n* = 3), and other sites (*n* = 13).

**TABLE 1:** Demographic and clinical characteristics.

Characteristics	ALL	PD‐L1 TPS 10%–49%	PD‐L1 TPS≥ 50%	*p*‐value
	*N* = 273	*N* = 127	*N* = 146	
Sex				.014
Male	157 (57.51%)	63 (49.61%)	94 (64.38%)	
Female	116 (42.49%)	64 (50.39%)	52 (35.62%)	
Age (years)				.045
≤60	137 (50.18%)	72 (56.69%)	65 (44.52%)	
>60	136 (49.82%)	55 (43.31%)	81 (55.48%)	
ECOG performance status				.059
0	3 (1.10%)	2 (1.57%)	1 (0.68%)	
1	254 (93.04%)	121 (95.28%)	133 (91.10%)	
2	16 (5.86%)	4 (3.15%)	12 (8.22%)	
Smoking status				.137
Never	181 (66.30%)	90 (70.87%)	91 (62.33%)	
Current or former	92 (33.70%)	37 (29.13%)	55 (37.67%)	
Histological subtype				.290
Adenocarcinoma	259 (94.87%)	119 (93.70%)	140 (95.89%)	
Squamous carcinoma	7 (2.56%)	5 (3.94%)	2 (1.37%)	
Adenosquamous carcinoma	5 (1.83%)	3 (2.36%)	2 (1.37%)	
Others	2 (0.73%)	0	2 (1.37%)	
Driver gene mutations				.078
EGFR	154 (56.41%)	81 (63.78%)	73 (50.00%)	
KRAS	50 (18.32%)	17 (13.39%)	33 (22.60%)	
ALK	22 (8.06%)	11 (8.66%)	11 (7.53%)	
Other types of gene mutations	47 (17.22%)	18 (14.17%)	29 (19.86%)	
PD‐L1 expression				.265
Primary tumor	202 (73.99%)	98 (77.17%)	104 (71.23%)	
Metastatic tumor	71 (26.01%)	29 (22.83%)	42 (28.77%)	
Brain metastasis				.513
Yes	104 (38.10%)	51 (40.16%)	53 (36.30%)	
No	169 (61.90%)	76 (59.84%)	93 (63.70%)	
Bone metastasis				.329
Yes	114 (41.76%)	57 (44.88%)	57 (39.04%)	
No	159 (58.24%)	70 (55.12%)	89 (60.96%)	
AJCC T stage				.789
T1	64 (23.44%)	26 (20.47%)	38 (26.03%)	
T2	86 (31.50%)	48 (37.80%)	38 (26.03%)	
T3	53 (19.41%)	23 (18.11%)	30 (20.55%)	
T4	70 (25.64%)	30 (23.62%)	40 (27.40%)	
AJCC N stage				.236
N0	41 (15.02%)	19 (14.96%)	22 (15.07%)	
N1	20 (7.33%)	9 (7.09%)	11 (7.53%)	
N2	81 (29.67%)	45 (35.43%)	36 (24.66%)	
N3	131 (47.99%)	54 (42.52%)	77 (52.74%)	

Abbreviations: AJCC, American Joint Committee on Cancer; ALK, anaplastic lymphoma kinase; ECOG, Eastern Cooperative Oncology Group; EGFR, epidermal growth factor receptor; KRAS, Kirsten rat sarcoma viral oncogene homolog; N, node; PD‐L1, programmed death‐ligand 1; T, tumor; TPS, tumor proportion score.

### 2.3 Statistical analysis

Progression‐free survival (PFS) and overall survival (OS) were calculated for all patients. Descriptive statistics were used to summarize baseline characteristics. Survival outcomes (PFS and OS) were estimated using the Kaplan–Meier method, and differences between groups were compared with the log‐rank test. A two‐sided *p*‐value of ≤.05 was considered statistically significant. Univariate and multivariate Cox proportional hazards analyses were performed to determine independent prognostic factors, based on which a predictive nomogram was developed by incorporating these significant variables.

All statistical analyses were performed using SPSS (version 24.0; IBM Corp., Armonk, NY, USA). Kaplan–Meier survival curves were plotted with GraphPad Prism (version 8.0; GraphPad Software, San Diego, CA, USA), and the nomogram was constructed using R software (version 4.4.0; R Foundation for Statistical Computing, Vienna, Austria).

Additional methodological details, including driver gene alteration assessment and supplementary statistical analyses, are provided in the Supplementary Materials and Methods.

## 3 RESULTS

### 3.1 Patient characteristics

**FIGURE 1:**
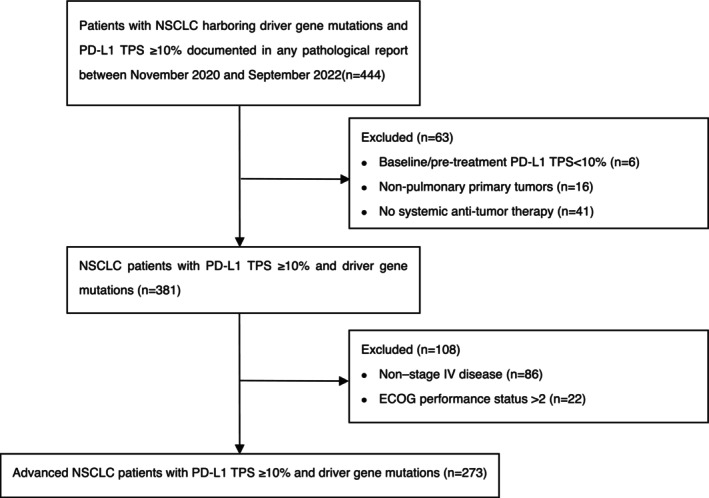
Flow chart depicting patient selection. ECOG, Eastern Cooperative Oncology Group; NSCLC, non‐small‐cell lung cancer; OS, overall survival; PD‐L1, programmed death‐ligand 1; TPS, tumor proportion score.

Initial screening identified 444 NSCLC patients with both driver gene mutations and a PD‐L1 TPS ≥10%. Based on the pre‐defined inclusion criteria (requiring pre‐treatment PD‐L1 expression), 6 patients were excluded due to a baseline PD‐L1 TPS <10%. After screening, 273 patients were ultimately included in the final analysis, as shown in Figure 1.

We analyzed 273 patients with stage IV NSCLC harboring confirmed driver gene mutations and PD‐L1 expression with a TPS ≥ 10%. The median follow‐up period was 23.8 months. The median age was 61 years (range, 27–87), with 157 patients (57.51%) male and 116 (42.49%) female. ECOG performance status was 0 in 3 patients (1.10%), 1 in 254 (93.04%), and 2 in 16 (5.86%). A history of current or past smoking was reported in 92 patients (33.70%). Most tumors were adenocarcinomas (*n* = 259, 94.87%), followed by squamous cell carcinoma (*n* = 7, 2.56%) (Table 1).

Among the 273 patients, 127 (46.52%) had PD‐L1 TPS of 10%–49%, and 146 (53.47%) had PD‐L1 TPS ≥50%. Driver mutations included EGFR (*n* = 154), KRAS (*n* = 50), ALK (*n* = 22), MET (*n* = 14), RET (*n* = 9), ROS1 (*n* = 9), BRAF V600E (*n* = 9), NRAS (*n* = 2), PIK3CA (*n* = 1), and HER2 (*n* = 3) (Supplementary Table 1).

### 3.2 Impact of PD‐L1 expression on prognosis in driver‐mutated NSCLC

**FIGURE 2:**
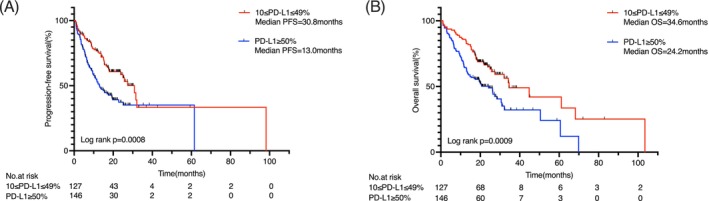
(A) OS and (B) PFS in patients with advanced driver gene‐positive NSCLC with PD‐L1 TPS 10%–49% or TPS ≥50. NSCLC, non‐small‐cell lung cancer; OS, overall survival; PD‐L1, programmed death‐ligand 1; PFS, progression‐free survival; TPS, tumor proportion score.

In the overall cohort, the median PFS was 13.8 months, and the median OS was 19.5 months. Patients with PD‐L1 TPS 10%–49% had significantly longer median PFS than those with TPS ≥50% (30.8 months vs. 13.0 months; *p* = .0008) (Figure 2A). Similarly, median OS was 34.6 months in the TPS 10%–49% group compared to 24.2 months in the TPS ≥50% group (*p* = .0009) (Figure 2B).

### 3.3 Impact of PD‐L1 expression on targeted therapy outcomes

**FIGURE 3:**
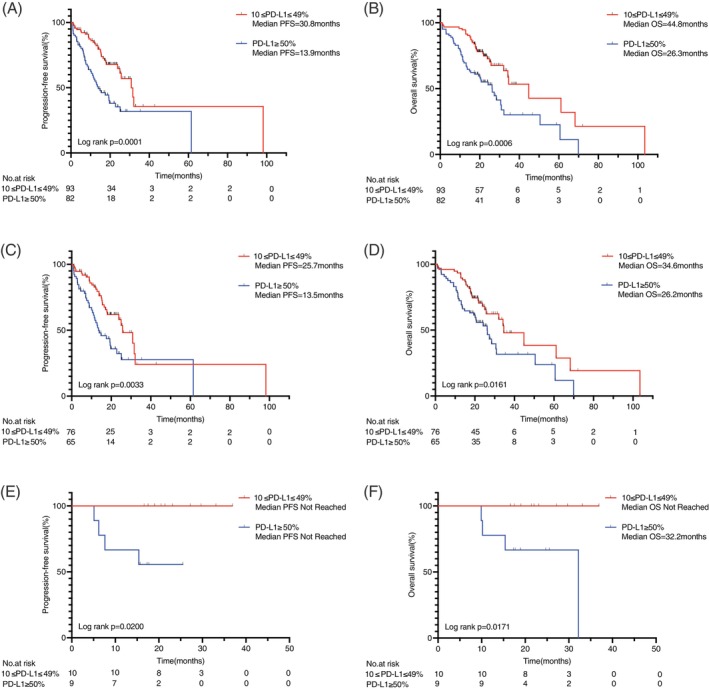
(A) PFS and (B) OS in all patients receiving TKI treatment with PD‐L1 TPS 10%–49% or TPS ≥50%. (C) PFS and (D) OS in patients receiving EGFR‐TKIs with PD‐L1 TPS 10%–49% or TPS ≥50%. (E) PFS and (F) OS in patients receiving ALK‐TKIs with PD‐L1 TPS 10%–49% or TPS ≥50%. ALK, anaplastic lymphoma kinase; EGFR, epidermal growth factor receptor; OS, overall survival; PD‐L1, programmed death‐ligand 1; PFS, progression‐free survival; TKI, tyrosine kinase inhibitor; TPS, tumor proportion score.

Among the 273 patients, 175 received first‐line TKI therapy, including those with EGFR (*n* = 141), ALK (*n* = 19), ROS1 (*n* = 6), MET (*n* = 5), RET (*n* = 3), and BRAF V600E (*n* = 1) alterations. Of these, 93 had PD‐L1 TPS 10%–49%, and 82 had TPS ≥50%. Median PFS was significantly longer in the 10%–49% group (30.8 months vs. 13.9 months; *p* = .0001) (Figure 3A), and median OS was also significantly prolonged (44.8 months vs. 26.3 months; *p* = .0006) (Figure 3B).

In patients with EGFR mutations, those with TPS 10%–49% (*n* = 76) had longer PFS than those with TPS ≥50% (*n* = 65) (25.7 months vs. 13.5 months; *p* = .0033) (Figure 3C) and also demonstrated superior OS (34.6 months vs. 26.2 months; *p* = .0161) (Figure 3D).

Among EGFR mutation subtypes, the most common was L858R (*n* = 75), followed by exon 19 deletions (*n* = 53). In patients with L858R mutations, no significant difference in PFS or OS was observed between PD‐L1 expression groups (Supplementary Figure 1A, B). However, among patients with exon 19 deletions, those with PD‐L1 TPS 10%–49% had significantly longer PFS (30.8 months vs. 13.5 months; *p* = .0103) and OS (44.8 months vs. 26.2 months; *p* = .0233) compared with those with TPS ≥50% (Supplementary Figure 1C, D).

Among patients with ALK rearrangements, 10 had PD‐L1 TPS 10%–49% and 9 had TPS ≥50%. Median PFS was significantly longer in the 10%–49% group (*p* = .0200) (Figure 3E). Median OS was not reached in the 10%–49% group, whereas it was 32.2 months in the TPS ≥50% group (*p* = .0171) (Figure 3F).

Of the 82 patients who progressed on first‐line TKI therapy and received second‐line treatment, 40 received another TKI and 42 received chemotherapy or other non‐TKI therapy. Median PFS was significantly shorter in those receiving second‐line TKI therapy compared with non‐TKI therapy (5.1 months vs. 20.7 months; *p* = .0172) (Supplementary Figure 1E), although OS did not differ significantly (*p* = .4507) (Supplementary Figure 1F). Among these, patients with PD‐L1 TPS ≥50% had significantly shorter OS than those with TPS 10%–49% (27.3 months vs. 61.1 months; *p* = .0182), while no significant difference was observed in PFS (Supplementary Figure 1G, H). Among the 40 patients who received second‐line TKI therapy, 14 (35.0%) were found to harbor identifiable resistance mutations and subsequently received matched targeted treatment. Subgroup analysis demonstrated that the median PFS was 6.7 months in patients with PD‐L1 TPS 10%–49% (*n* = 7) and 7.1 months in those with PD‐L1 TPS ≥50% (*n* = 7), with no statistically significant difference between the two groups (*p* = .6250). Similarly, the median OS was 68.2 months and 28.4 months, respectively, and the difference was not statistically significant (*p* = .2619) (Supplementary Figure 1I, J).

### 3.4 Impact of PD‐L1 expression on immunotherapy outcomes

**FIGURE 4:**
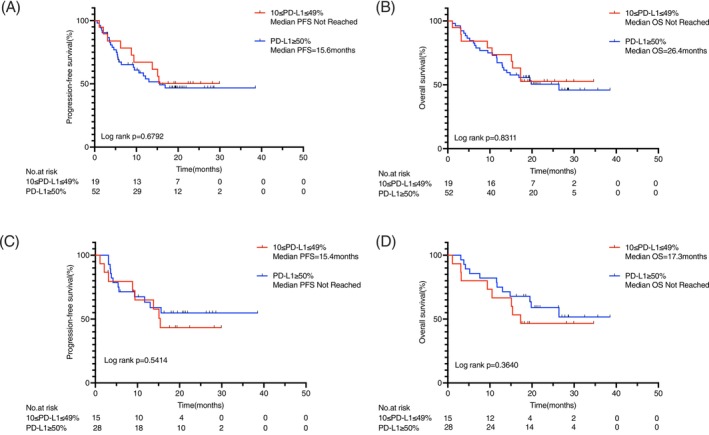
(A) PFS and (B) OS in all patients receiving immunotherapy with PD‐L1 TPS 10%–49% or TPS ≥50%. (C) PFS and (D) OS in KRAS‐mutated patients receiving immunotherapy with PD‐L1 TPS 10%–49% or TPS ≥50%. KRAS, Kirsten rat sarcoma viral oncogene homolog; OS, overall survival; PD‐L1, programmed death‐ligand 1; PFS, progression‐free survival; TPS, tumor proportion score.

Seventy‐one patients received first‐line immunotherapy. These included individuals with EGFR (*n* = 10), KRAS (*n* = 43), BRAF V600E (*n* = 6), MET (*n* = 6), RET (*n* = 2), ROS1 (*n* = 2), and NRAS (*n* = 2) mutations. Among them, 19 had PD‐L1 TPS 10%–49% and 52 had TPS ≥50%. No significant differences were observed in either PFS (*p* = .6792) or OS (*p* = .8311) between the two PD‐L1 groups (Figure 4A,B).

Among the 50 patients with KRAS mutations, 43 received first‐line immunotherapy and 7 received alternative therapies. Those receiving immunotherapy had significantly longer PFS (not reached vs. 4.4 months; *p* <.0001) and OS (26.4 months vs. 6.9 months; *p*<.0001) (Supplementary Figure 2A,B). Notably, within the KRAS‐immunotherapy cohort, no significant differences in PFS (*p* = .5414) or OS (*p* = .3640) were seen between PD‐L1 expression subgroups (Figure 4C,D).

In a subgroup of 82 patients receiving second‐line treatment after TKI resistance, 35 received immunotherapy and 47 received chemotherapy or other therapies. No significant differences were found in either PFS (*p* = .9266) or OS (*p* = .8693) between the two strategies (Supplementary Figure 2C, D). Among the 35 patients receiving second‐line immunotherapy, most had EGFR mutations (*n* = 32), and 2 had MET alterations. PD‐L1 TPS 10%–49% was observed in 16 patients, and TPS ≥50% in 19. No statistically significant differences in PFS (*p* = .2137) or OS (*p* = .0773) were observed between the two groups (Supplementary Figure 2E, F).

### 3.5 Construction and validation of the nomogram model

**FIGURE 5:**
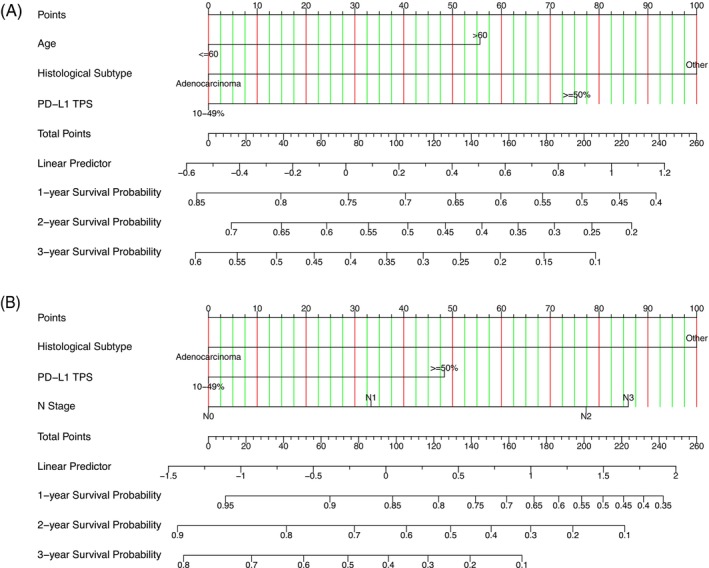
(A) Nomogram to estimate the risk in the overall study population. (B) Nomogram to estimate the risk in patients with EGFR mutations. AJCC, American Joint Committee on Cancer; EGFR, epidermal growth factor receptor; N, node; PD‐L1, programmed death‐ligand 1; TPS, tumor proportion score.

In the entire cohort, multivariate analysis (Supplementary Table 2) demonstrated that high PD‐L1 expression was identified as an independent unfavorable prognostic factor. We attempted to construct a nomogram to predict the 1‐year, 2‐year, and 3‐year OS of patients with driver gene mutations. The model incorporated three independent prognostic factors associated with OS, including age, histological subtype, and PD‐L1 expression status. The effectiveness of the nomogram was validated using calibration curves (Figure 5A and Supplementary Figure 3A–C). The concordance index (C‐index) for the predictive model was 0.639. The calibration curves of the nomogram showed that the predicted values and the actual observed values had good agreement in certain intervals.

Given that more than half of the patients in the study cohort harbored EGFR mutations, we also developed a nomogram to predict the 1‐year, 2‐year, and 3‐year OS for patients receiving EGFR‐TKI therapy. Based on prognostic factors identified from multivariate analysis of EGFR‐TKI–treated patients (Supplementary Table 3), the nomogram included variables relevant to this population, such as histological subtype, PD‐L1 expression status, and N stage (Figure 5B). The resulting model had a C‐index of 0.621, and calibration curves demonstrated good agreement between predicted and observed survival in key prediction intervals (Supplementary Figure 3D–F).

## 4 DISCUSSION

Previous studies have primarily focused on the prognostic impact of PD‐L1 expression in patients with single driver mutations,^16^, ^17^ and others have examined its association with multiple oncogenic alterations.^22^, ^23^ In this study, we sought to assess the prognostic and therapeutic implications of PD‐L1 expression in a comprehensive cohort of patients with stage IV NSCLC harboring a variety of driver mutations.

Our analysis demonstrated that patients with PD‐L1 tumor proportion scores (TPS) of 10%–49% had significantly longer median PFS and OS than those with TPS ≥50%. These findings suggest that higher PD‐L1 expression may have distinct prognostic significance in the context of oncogene‐driven NSCLC.

Among patients who received first‐line TKI therapy—including those with EGFR, ALK, ROS1, MET, RET, and BRAF V600E mutations—we observed that higher PD‐L1 expression was consistently associated with poorer survival outcomes (30.8 months vs. 13.9 months; mOS: 44.8 months vs. 26.3 months). These results support the hypothesis that elevated PD‐L1 expression may be an adverse prognostic factor for patients undergoing targeted therapy, especially in those with EGFR mutations. Our findings align with prior studies that demonstrated inferior survival among EGFR‐mutated NSCLC patients with high PD‐L1 expression receiving EGFR‐TKIs.^14^, ^17^

One proposed mechanism for this observation is that PD‐L1 may promote primary resistance to EGFR‐TKIs via the MAPK/ERK signaling pathway and autophagy induction in EGFR‐mutant lung adenocarcinoma cells.^24^ Moreover, we found that the impact of PD‐L1 expression varied by EGFR mutation subtype. Among patients with exon 19 deletions, those with PD‐L1 TPS 10%–49% experienced significantly longer PFS and OS compared to those with TPS ≥50%. However, no such difference was observed in patients with L858R mutations. These data suggest that PD‐L1 expression may differentially modulate cellular proliferation, apoptosis, and autophagy depending on EGFR mutation subtype—an area warranting further mechanistic exploration.

Similar trends were noted among ALK‐rearranged patients, where high PD‐L1 expression was associated with poorer outcomes following ALK‐TKI therapy. This is consistent with prior evidence indicating that elevated PD‐L1 levels may be linked to inferior responses to second‐generation ALK inhibitors in advanced ALK‐positive NSCLC.^15^, ^16^ These findings underscore the potential predictive value of PD‐L1 expression in guiding targeted therapy decisions beyond EGFR mutations. We further evaluated treatment patterns and outcomes after progression on first‐line TKI therapy. Patients who received second‐line non‐TKI treatment achieved a significantly longer median PFS than those who continued TKI therapy (20.7 vs. 5.1 months, *p* = .0172). Although prior studies have suggested potential benefits of sequential TKI strategies in EGFR‐ or ALK‐mutant NSCLC,^12^, ^25^ our results did not fully replicate these findings. This discrepancy may be partly explained by the limited sample size and the heterogeneity of second‐line TKI use, as only a subset of patients received molecularly matched targeted agents based on identifiable resistance mutations. In a small subgroup of patients treated with resistance mutation–matched therapy, no significant differences in PFS or OS were observed between PD‐L1 TPS 10%–49% and ≥50% groups, suggesting that PD‐L1 expression may have limited prognostic relevance in the context of well‐defined resistance mechanisms. However, given the exploratory nature and limited sample size, these findings should be interpreted with caution and warrant validation in larger prospective studies incorporating comprehensive post‐resistance molecular profiling.

While PD‐L1 expression has been firmly established as a predictive biomarker for response to ICIs in patients with wild‐type NSCLC,^4^, ^7^, ^8^ our findings suggest that its predictive value may not extend to patients with oncogenic driver mutations. Among patients receiving first‐line immunotherapy, we observed no significant differences in PFS or OS between PD‐L1 TPS 10%–49% and ≥50% subgroups. This may reflect the biological complexity of oncogene‐driven tumors, where PD‐L1 expression alone may not adequately capture immune responsiveness.^26^

It is important to note that our cohort included some patients for whom first‐line immunotherapy would not typically be indicated under current treatment guidelines. The inclusion of such patients could have influenced outcomes, as treatment selection may not have been optimal in all cases.^27^, ^28^ To further elucidate the relationship between PD‐L1 expression and immunotherapy efficacy, we analyzed a subgroup of patients with KRAS mutations. Consistent with existing literature, KRAS‐mutated NSCLC was associated with favorable responses to ICIs.^29^ However, within this subgroup, we found no significant survival difference based on PD‐L1 expression levels, suggesting that PD‐L1 may not independently predict benefit from immunotherapy in KRAS‐mutant patients.

Our results are consistent with reports from several large studies evaluating ICIs across NSCLC patients with diverse molecular alterations (e.g., ALK, ROS1, BRAF V600E, MET, RET, NTRK, KRAS, HER2), in which PD‐L1 expression was not consistently associated with clinical benefit.^30^, ^31^ This underscores the limitations of PD‐L1 as a solitary biomarker and suggests that its predictive utility may be context‐dependent. In our analysis of second‐line treatment following TKI resistance, no significant difference in PFS or OS was observed between patients receiving immunotherapy and those receiving chemotherapy. Likewise, PD‐L1 expression levels did not significantly influence outcomes in this setting.

Given that most patients in our second‐line immunotherapy cohort had EGFR mutations, we compared our findings to prior studies of ICI efficacy in EGFR‐TKI–resistant NSCLC. One study identified PD‐L1 TPS as an independent predictor of OS in this population,^32^ though our results did not replicate this association. It is possible that limited sample size, treatment heterogeneity, or unmeasured confounding factors may have attenuated the predictive value of PD‐L1 in our analysis. These limitations underscore the need for more comprehensive biomarker strategies that incorporate multiple molecular and immune‐related indicators to improve treatment selection.^33^, ^34^

Emerging biomarkers under investigation—such as microsatellite instability, tumor mutational burden, and T‐cell‐inflamed gene expression profiles—may offer additional predictive insight when used in combination with PD‐L1.^35^, ^36^, ^37^

The results of this study suggest that high PD‐L1 expression is an independent unfavorable prognostic factor in patients with driver gene mutations as well as those receiving EGFR‐TKI therapy. Survival analysis consistently demonstrated that high PD‐L1 expression is associated with shorter PFS and OS. Given the strong correlation between PD‐L1 expression and survival outcomes, we constructed nomogram models for both the overall population and the EGFR‐TKI subgroup.

Using the nomogram, we integrated multiple independent prognostic factors—such as age, pathological features, and biomarkers—to bridge statistical modeling with clinical application, offering personalized risk predictions for patients.^38^ By incorporating PD‐L1 and other independent prognostic factors, we quantified the weights of each variable to predict survival probabilities. The results indicated that the predicted probabilities aligned well with actual outcomes in certain intervals, and the model demonstrated significant clinical reference value at specific time points. This provides guidance for the personalized management of patients with driver gene‐positive NSCLC.

Although the C‐index of the model (C‐index = 0.639 for the overall population; C‐index = 0.621 for the EGFR‐TKI subgroup) did not reach the ideal threshold (C‐index >0.7), the model establishes a basic framework for risk prediction based on clinical characteristics. In the future, the predictive accuracy of the model could be enhanced by including a larger sample size, more comprehensive prognostic factors, and dynamic variables, thereby improving its applicability in clinical decision‐making.

This study has several limitations. First, it was a single‐center retrospective analysis, with the study population predominantly harboring EGFR, KRAS, or ALK alterations, while rare driver mutations were underrepresented. In addition, patients with PD‐L1 TPS 1%–9% or PD‐L1‐negative tumors were not systematically included, which may limit the generalizability of our findings across the full spectrum of PD‐L1 expression. Second, the cohort consisted mainly of patients with lung adenocarcinoma, and other histological subtypes were insufficiently represented, limiting the applicability of our conclusions to non‐adenocarcinoma NSCLC. Finally, the predictive model was developed based on a relatively limited sample size and a restricted number of prognostic variables. Larger multicenter prospective studies incorporating broader molecular and histological subtypes, standardized PD‐L1 assessment, and more comprehensive clinical data are warranted to further validate and refine these findings.

In summary, this study explores the potential relevance of driver gene mutation status and PD‐L1 expression for prognostic assessment and therapeutic stratification among patients with advanced NSCLC and PD‐L1 TPS ≥10%. Within this population, higher PD‐L1 expression appeared to be associated with poorer outcomes following TKI therapy, particularly in patients harboring EGFR or ALK alterations. In contrast, PD‐L1 expression did not show a consistent association with immunotherapy outcomes in this subgroup. Among patients with KRAS mutations, immunotherapy was associated with improved survival irrespective of PD‐L1 expression levels (10%–49% or ≥50%) in this cohort. Collectively, these findings highlight the complexity of biomarker interpretation in oncogene‐driven NSCLC and underscore the need for more nuanced, biomarker‐informed approaches to optimize treatment strategies.

## AUTHOR CONTRIBUTIONS

**Xiaoxiao Fan:** Conceptualization; methodology; writing – original draft; data curation; writing – review and editing. **Chenxi Wei:** Data curation; writing – review and editing. **Minjun Rong:** Validation; writing – review and editing. **Shengnan Wang:** Data curation; validation; writing – review and editing. **Jiaying Wang:** Writing – review and editing. **Xiaohan Wang:** Data curation; writing – review and editing. **Xiao Han:** Conceptualization; methodology; writing – review and editing. **Xue Meng:** Conceptualization; methodology; funding acquisition.

## FUNDING INFORMATION

This article was supported by National Natural Science Foundation of China (82172720), CSCO‐Nav HER2‐related Solid Tumors Research Foundation (Y‐2022HER2AZMS‐0291), Natural Science Foundation of Shandong Province (ZR2023LSW024) and Wu Jieping Medical Foundation (320.6750.2022‐17‐46).

## CONFLICT OF INTEREST STATEMENT

All authors certify that they have no affiliations with or involvement in any organization or entity with any financial interest or non‐financial interest in the subject matter or materials discussed in this manuscript.

## ETHICS STATEMENT

This study was granted exemption by the ethics committee of Shandong Cancer Hospital and Institute Reviewer Board (approval no. SDTHEC202506026).

## Supporting information


**Supplementary Table 1.** Distribution of driver gene mutations in the study cohort (*N* = 273).
**Supplementary Table 2**. Cox univariate and multivariate analysis of overall survival in patients with gene mutations (*N* = 273).
**Supplementary Table 3**. Cox univariate and multivariate analysis of overall survival in patients receiving EGFR‐TKI treatment (*N* = 141).
**Supplementary Figure 1**. (A) PFS and (B) OS in patients with the 21L858R mutation receiving EGFR‐TKIs with PD‐L1 TPS 10%–49%.
**Supplementary Figure 2**. (A) PFS and (B) OS in KRAS‐mutated patients receiving immunotherapy or non‐immunotherapy. (C) PFS.
**Supplementary Figure 3**. (A) 1‐year, (B) 2‐year, and (C) 3‐year OS calibration curves of the nomogram in the overall study population.

## Data Availability

All data generated or analyzed during this study are included in this published article and its supplementary information files. Further information is available from the corresponding author upon request.
