# Supplementary material for: Clinical implications of PD‐L1 expression in oncogene‐driven NSCLC: Differential responses to targeted agents and immune checkpoint inhibitors
Source: Int J Cancer. 2026 Mar 23;159(1):269–79. doi: 10.1002/ijc.70413 (PMC13140019; doi:10.1002/ijc.70413)
Supplement: Supplementary file 1 — Supplementary Table 1. Distribution of driver gene mutations in the study cohort (N = 273). Supplementary Table 2. Cox univariate and multivariate analysis of overall survival in patients with gene mutations (N = 273). Supplementary Table 3. Cox univariate and multivariate analysis of overall survival in patients receiving EGFR‐TKI treatment (N = 141). Supplementary Figure 1. (A) PFS and (B) OS in patients with the 21L858R mutation receiving EGFR‐TKIs with PD‐L1 TPS 10%–49%. Supplementary Figure 2. (A) PFS and (B) OS in KRAS‐mutated patients receiving immunotherapy or non‐immunotherapy. (C) PFS. Supplementary Figure 3. (A) 1‐year, (B) 2‐year, and (C) 3‐year OS calibration curves of the nomogram in the overall study population. [file IJC-159-269-s001.pdf]

**Title: Clinical Implications of PD-L1 Expression in Oncogene-Driven NSCLC: Differential Responses to Targeted Agents and Immune Checkpoint Inhibitors**

**Authors:Xiaoxiao Fan, Chenxi Wei, Minjun Rong, Shengnan Wang, Jiaying Wang, Xiaohan Wang, Xiao Han, Xue Meng**

## Contents

**List of Abbreviations** ........ 2

**Supplementary Materials & Methods** ........ 2

**Supplementary Table1** ........ 3

**Supplementary Table2** ........ 4

**Supplementary Table3** ........ 6

**Supplementary Figure 1** ........ 7

**Supplementary Figure 2** ........ 8

**Supplementary Figure 3** ........ 9

**List of Abbreviations**

AJCC: American Joint Committee on Cancer;ALK: Anaplastic Lymphoma Kinase; ARMS-PCR: amplification refractory mutation system polymerase chain reaction; BRAF: v-raf murine sarcoma viral oncogene homolog B1;ECOG: Eastern Cooperative Oncology Group;EGFR: Epidermal Growth Factor Receptor;EGFR 19Del: EGFR exon 19 deletion;EGFR 21L858R: EGFR exon 21 L858R point mutation; HER2: Human Epidermal Growth Factor Receptor 2;ICI: Immune checkpoint inhibitor; KRAS: Kirsten rat sarcoma viral oncogene homolog;KRAS-G12C: KRAS codon 12 glycine to cysteine substitution;MET: Mesenchymal–epithelial transition factor;METamp: MET gene amplification;METex14: MET exon 14 skipping mutation;N: Node; NGS: next-generation sequencing;NRAS: Neuroblastoma RAS viral oncogene homolog;NSCLC: Non–small-cell lung cancer;OS: Overall survival;PD-L1: Programmed death-ligand 1;PFS: Progression-free survival;PIK3CA: Phosphatidylinositol-4,5-bisphosphate 3-kinase catalytic subunit alpha; RET:Rearranged during transfection;ROS1: c-ros oncogene 1;T: Tumor;TKI: Tyrosine kinase inhibitor;TPS: Tumor proportion score.

**Supplementary Materials & Methods**

**Assessment of Driver Gene Alterations**

Driver gene alterations were identified using routine molecular diagnostic assays applied in clinical practice during the study period. Detection methods included amplification refractory mutation system polymerase chain reaction (ARMS-PCR) and next-generation sequencing (NGS), depending on tissue availability and testing period. All molecular analyses were performed in certified clinical laboratories following standardized quality control procedures.

**Statistical Analysis for Supplementary Tables and Figures**

Statistical analyses for Supplementary Tables 1-3 and Supplementary Figures 1-3 were performed using the same software and statistical approaches as described in the main manuscript: SPSS (version 24.0; IBM Corp., Armonk, NY, USA), GraphPad Prism (version 8.0; GraphPad Software, San Diego, CA, USA), and R software (version 4.4.0). The distribution of driver gene mutations (including EGFR, KRAS, ALK, MET, RET, ROS1, BRAF V600E, NRAS, PIK3CA, and HER2) across PD-L1 expression subgroups (TPS 10-49% vs. ≥50%) was summarized using descriptive statistics. Categorical variables (mutation subtypes) were presented as counts and percentages.

Survival outcomes, including progression-free survival (PFS) and overall survival (OS), were estimated using the Kaplan–Meier method, and differences between groups were compared using the log-rank test. Cox proportional hazards regression analyses were then performed to identify prognostic factors associated with survival outcomes. Univariate Cox proportional hazards analyses were conducted initially, followed by multivariate models incorporating variables of potential clinical relevance to determine independent prognostic factors.All statistical tests were two-sided, and a P value ≤0.05 was considered statistically significant.

Calibration curves were generated to assess the agreement between nomogram-predicted and observed survival probabilities.Internal validation was performed using 1,000 bootstrap resamples, and calibration was assessed at 1, 2, and 3 years. A calibration curve close to the 45-degree line indicated good agreement between predicted and observed outcomes.

**Supplementary Table1.** Distribution of driver gene mutations in the study cohort (N=273).

| Driver gene mutations | ALL | PD-L1 10-49% | PD-L1≥50% |
|---|---|---|---|
| | N=273 | N=127 | N=146 |
| EGFR | | | |
| EGFR 19Del | 57 (20.88%) | 35 (27.56%) | 22 (15.07%) |
| EGFR 21L858R | 78 (28.57%) | 38 (29.92%) | 40 (27.40%) |
| Uncommon EGFR mutations | 19 (6.96%) | 8 (6.30%) | 11 (7.53%) |
| KRAS | | | |
| KRAS-G12C | 16 (5.86%) | 5 (3.94%) | 11 (7.53%) |
| Non-KRAS G12C | 34 (12.45%) | 12 (9.45%) | 22 (15.07%) |
| MET | | | |
| METex14 | 12 (4.40%) | 4 (3.15%) | 8 (5.48%) |
| METamp | 2 (0.73%) | 0 | 2 (1.37%) |
| ALK | 22 (8.06%) | 11 (8.66%) | 11 (7.53%) |
| RET | 9 (3.30%) | 4 (3.15%) | 5 (3.42%) |
| ROS1 | 9 (3.30%) | 5 (3.94%) | 4 (2.74%) |
| BRAF V600E | 9 (3.30%) | 2 (1.57%) | 7 (4.79%) |
| NRAS | 2 (0.73%) | 0 | 2 (1.37%) |
| PIK3CA | 1 (0.37%) | 1 (0.79%) | 0 |
| HER2 | 3 (1.10%) | 2 (1.57%) | 1 (0.68%) |

**Supplementary Table2.** Cox univariate and multivariate analysis of overall survival in patients with gene mutations(N=273).

| Characteristics | Univariate analysis | | Multivariate analysis | |
|---|---|---|---|---|
| | HR (95%CI) | P | HR (95%CI) | P |
| Age | | | | |
| ≤60 | Reference | | Reference | |
| ＞60 | 1.65 (1.17-2.33) | 0.004 | 1.48 (1.05-2.10) | 0.027 |
| Sex | | | | |
| Male | Reference | | | |
| Female | 0.75 (0.53-1.06) | 0.105 | | |
| ECOG performance status | | | | |
| 0 | Reference | | | |
| 1 | 0.54 (0.13-2.21) | 0.394 | | |
| 2 | 0.90 (0.20-4.12) | 0.891 | | |
| Smoking status | | | | |
| Never | Reference | | | |
| Current or former | 1.08 (0.75-1.54) | 0.687 | | |
| Histological subtype | | | | |
| Adenocarcinoma | Reference | | Reference | |
| Other | 2.18 (1.14-4.17) | 0.018 | 2.02 (1.05-3.88) | 0.034 |
| Driver gene mutations | | | | |
| EGFR | Reference | | | |
| KRAS | 1.46 (0.94-2.27) | 0.096 | | |
| ALK | 0.56 (0.24-1.30) | 0.177 | | |
| Other | 1.44 (0.91-2.30) | 0.121 | | |
| PD-L1 TPS | | | | |
| 10-49% | Reference | | Reference | |
| ⩾50% | 1.79 (1.26-2.55) | 0.001 | 1.70 (1.20-2.43) | 0.003 |
| PD-L1 expression | | | | |
| Primary lesion | Reference | | | |
| Metastatic lesion | 0.86 (0.58-1.28) | 0.455 | | |
| Brain metastasis | | | | |
| No | Reference | | | |
| Yes | 0.90 (0.63-1.28) | 0.540 | | |
| Bone metastasis | | | | |
| No | Reference | | | |
| Yes | 1.34 (0.96-1.89) | 0.090 | | |
| AJCC T stage | | | | |
| T1 | Reference | | | |
| T2 | 1.28 (0.79-2.07) | 0.317 | | |
| T3 | 1.35 (0.78-2.33) | 0.279 | | |
| T4 | 1.47 (0.89-2.44) | 0.135 | | |

**Supplementary Table 2 (continued)**

| Characteristics | Univariate analysis | | Multivariate analysis | |
| --- | --- | --- | --- | --- |
| | **HR (95%CI)** | **P** | **HR (95%CI)** | **P** |
| AJCC N stage | | | | |
| N0 | Reference | | | |
| N1 | 1.20 (0.55-2.61) | 0.645 | | |
| N2 | 1.34 (0.77-2.32) | 0.304 | | |
| N3 | 1.29 (0.76-2.18) | 0.345 | | |

**Supplementary Table3.**Cox univariate and multivariate analysis of overall survival in patients receiving EGFR-TKI treatment(N=141).

| Characteristics | Univariate analysis | | Multivariate analysis | |
|---|---|---|---|---|
| | HR (95%CI) | P | HR (95%CI) | P |
| Age | | | | |
| ≤60 | Reference | | | |
| ＞60 | 1.24 (0.77-2.00) | 0.377 | | |
| Sex | | | | |
| Male | Reference | | | |
| Female | 0.83 (0.51-1.34) | 0.449 | | |
| ECOG performance status | | | | |
| 1 | Reference | | | |
| 2 | 1.37 (0.55-3.42) | 0.501 | | |
| Smoking status | | | | |
| Never | Reference | | | |
| Current or former | 0.85 (0.49-1.48) | 0.577 | | |
| Histological subtype | | | | |
| Adenocarcinoma | Reference | | Reference | |
| Other | 2.95 (1.26-6.90) | 0.013 | 3.55 (1.47-8.57) | 0.005 |
| PD-L1 TPS | | | | |
| 10-49% | Reference | | Reference | |
| ≥50% | 1.79 (1.11-2.90) | 0.018 | 1.80 (1.10-2.92) | 0.019 |
| EGFR gene mutations | | | | |
| Classical mutations | Reference | | | |
| Other | 2.16 (1.07-4.39) | 0.033 | | |
| PD-L1 expression | | | | |
| Primary lesion | Reference | | | |
| Metastatic lesion | 0.93 (0.53-1.65) | 0.807 | | |
| Brain metastasis | | | | |
| No | Reference | | | |
| Yes | 1.34 (0.83-2.17) | 0.237 | | |
| Bone metastasis | | | | |
| No | Reference | | | |
| Yes | 1.16 (0.72-1.88) | 0.548 | | |
| AJCC T stage | | | | |
| T1 | Reference | | | |
| T2 | 1.50 (0.73-3.09) | 0.270 | | |
| T3 | 1.34 (0.58-3.12) | 0.497 | | |
| T4 | 1.41 (0.64-3.09) | 0.388 | | |
| AJCC N stage | | | | |
| N0 | Reference | | Reference | |
| N1 | 1.34 (0.39-4.65) | 0.640 | 1.46 (0.42-5.14) | 0.552 |
| N2 | 2.27 (0.98-5.26) | 0.056 | 2.45 (1.02-5.88) | 0.045 |
| N3 | 2.37 (1.04-5.40) | 0.040 | 2.88 (1.22-6.79) | 0.016 |

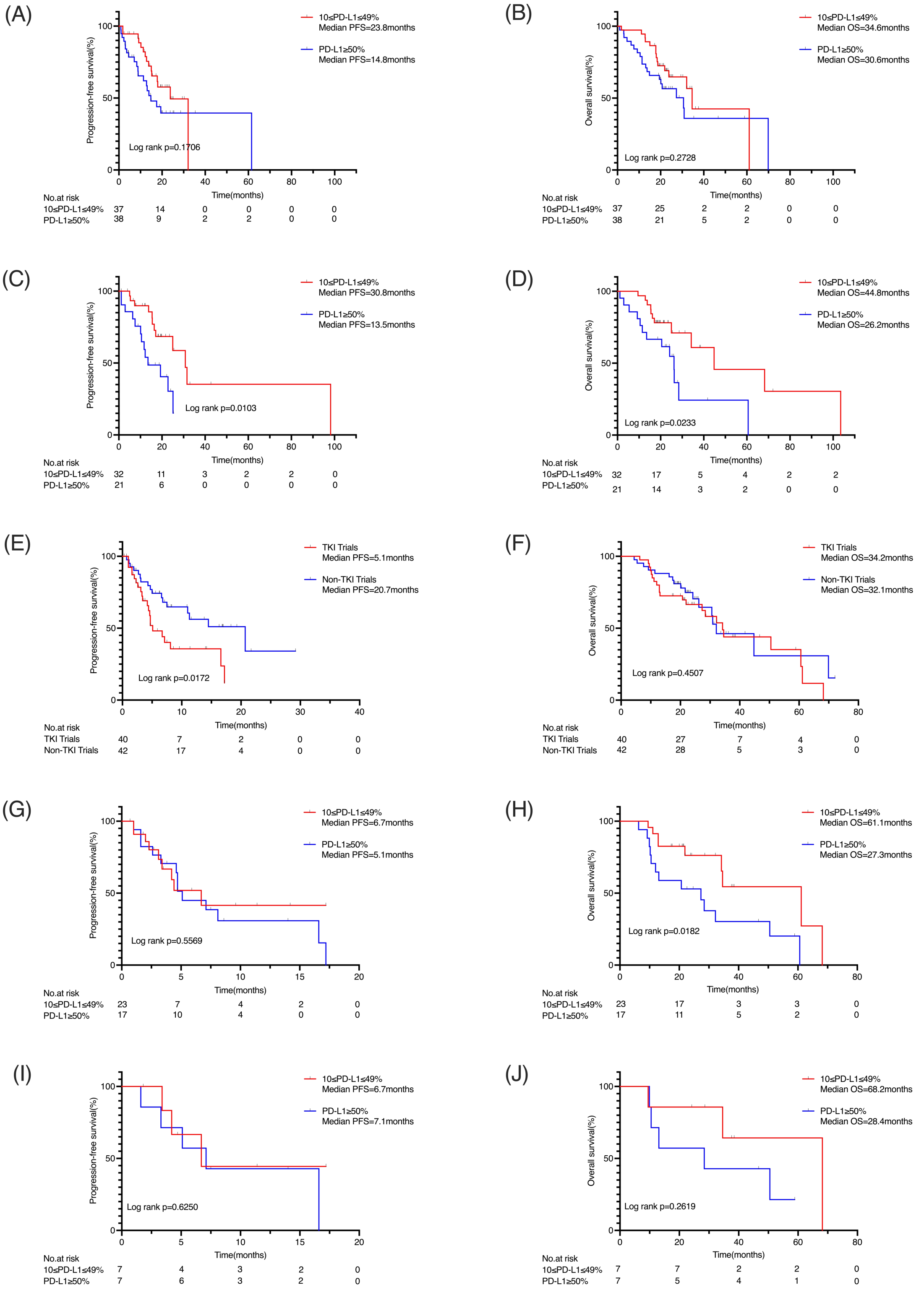


**Supplementary Figure 1**.(A)PFS and (B) OS in patients with the 21L858R mutation receiving EGFR-TKIs with PD-L1 TPS 10-49% or TPS≥50%. (C)PFS and (D) OS in patients with the 19DEL mutation receiving EGFR-TKIs with PD-L1 TPS 10-49% or TPS≥50%. (E)PFS and (F) OS in patients choosing targeted or non-targeted therapy as second-line treatment. (G)PFS and (H) OS in patients receiving targeted therapy as second-line treatment with PD-L1 TPS 10-49% or TPS≥50%. (I) PFS and (J) OS in patients receiving matched targeted therapy as second-line treatment with PD-L1 TPS 10–49% or TPS ≥50%.

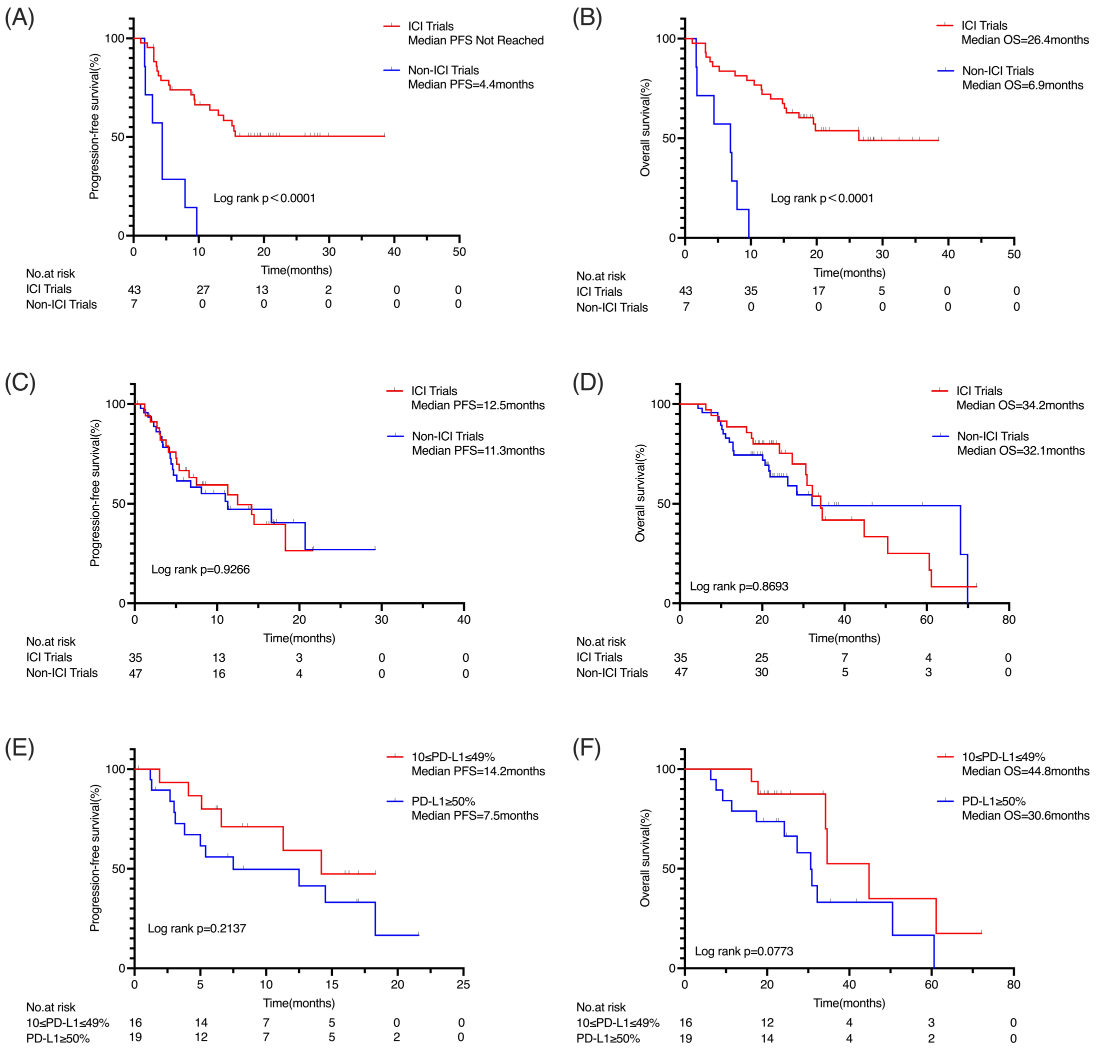


**Supplementary Figure 2**.(A)PFS and (B) OS in KRAS-mutated patients receiving immunotherapy or non-immunotherapy. (C)PFS and (D) OS in patients choosing immunotherapy or non-immunotherapy as second-line treatment. (E)PFS and (F) OS in patients receiving second-line immunotherapy with PD-L1 TPS 10-49% or TPS≥50%.

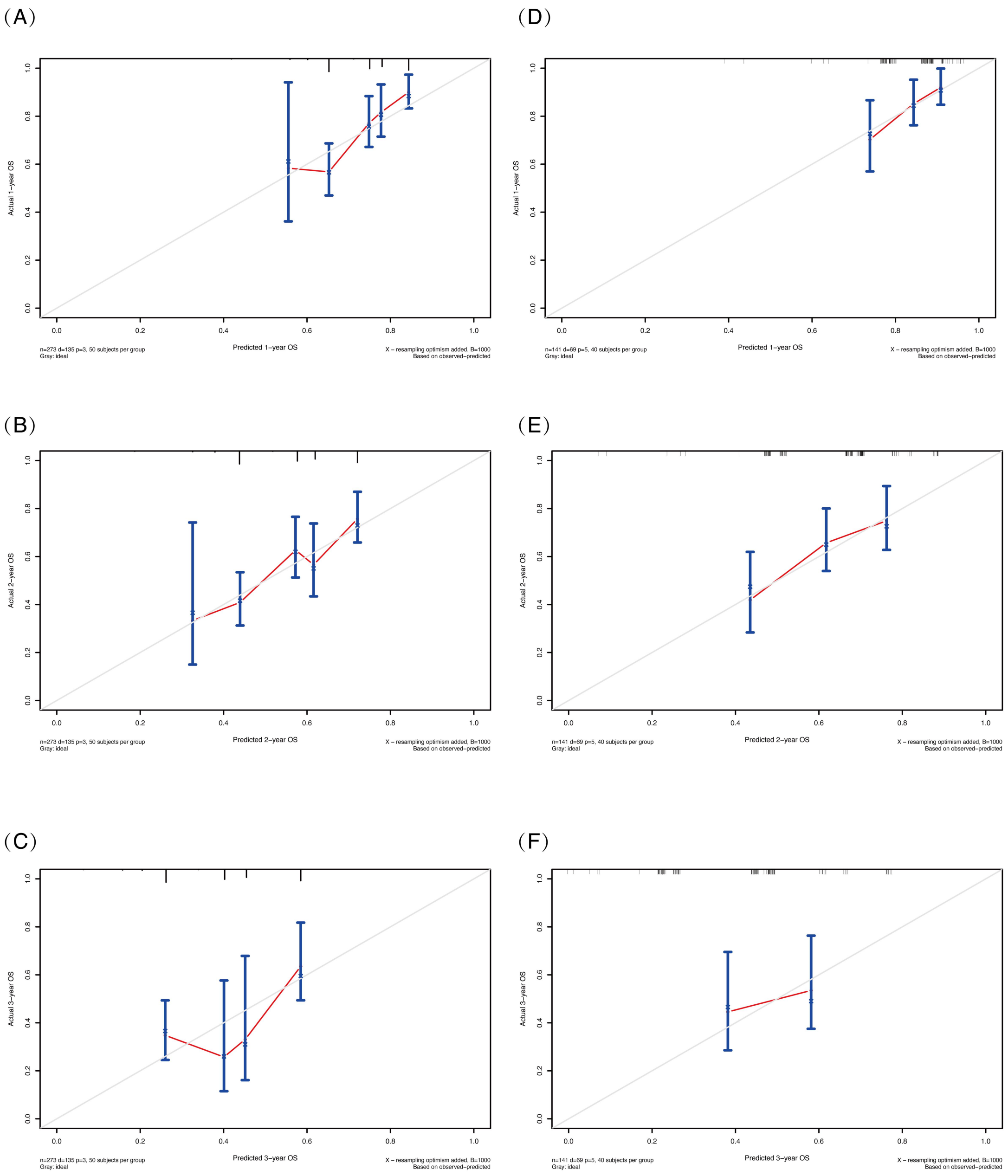


**Supplementary Figure 3.**(A) 1-year, (B) 2-year, and (C) 3-year OS calibration curves of the nomogram in the overall study population. (D)1-year, (E) 2-year, and (F) 3-year OS calibration curves of the nomogram in EGFR-mutated patients.
